# FDA guidance for next generation sequencing-based testing: balancing regulation and innovation in precision medicine

**DOI:** 10.1038/s41525-018-0067-2

**Published:** 2018-10-03

**Authors:** Frank Luh, Yun Yen

**Affiliations:** 1 Sino-American Cancer Foundation, Temple City, CA USA; 2 PhD Program of Cancer Biology and Drug Discovery, TMU Research Center of Cancer Translational Medicine, Taipei Medical University, Taipei, Taiwan

On 12 April 2018, the Food and Drug Administration (FDA) finalized a guidance document, Considerations for Design, Development, and Analytical Validation of Next Generation Sequencing (NGS)-Based In Vitro Diagnostics (IVDs) Intended to Aid in the Diagnosis of Suspected Germline Diseases, in efforts to accelerate the establishment of a regulatory approach for next generation sequencing (NGS) testing.^1^ While the FDA’s guidance is a step forward towards integrating NGS into clinical practice, the document has rekindled the debate about the FDA’s authority despite existing rules already enforced by Centers for Medicare and Medicaid Services (CMS). The FDA’s proposed framework also raises concerns as to whether it effectively protects consumer safety or threatens scientific discovery and innovation. As questions about regulation, statutory power, and legislation reform remain, the FDA and healthcare leaders must consider logistical and stakeholder challenges, if they seek to transform clinical care with NGS technology.

## NGS technology and FDA response

In the past decade, the cost of sequencing a whole genome has dropped 1000-fold,^2^ and the number of genetic tests has risen to more than 55,000 for over 11,000 conditions.^3^ Rapid adoption of NGS technology in medicine has lead to the identification and curation of novel genetic variants that promise to improve diagnostic accuracy and reduce unnecessary healthcare costs.^4^ Without question, we have witnessed the greatest impact of genomics in oncology and cancer therapy. The diagnosis and management of several types of cancer – Hodgkin’s lymphoma, breast cancer, and chronic myeloid leukemia - have made remarkable advances thanks to DNA sequencing technology.^5^ NGS has also benefitted other fields like cardiovascular medicine. The TRITON-TIMI 38 trial highlighted the possibility of adverse clinical outcomes when patients with cytochrome P-450 genetic variants are treated with clopidogrel.^6^

As science and medicine continue to develop therapies tailored to specific features of a patient’s genome, the FDA has taken steps to monitor the development, safety, and efficaciousness of NGS-testing. Historically, the FDA has exercised enforcement discretion of laboratory-developed tests (LDTs), which the FDA defines as “an in vitro diagnostic (IVD) device that is intended for clinical use and designed, manufactured, and used within a single laboratory.”^7^ The agency took the position that LDTs were “relatively simple lab tests and generally available on limited basis.”^8^ As a result, these tests were not subject to the same FDA quality or validity standards that applied to diagnostic tests made by medical device manufacturers. In recent years, labs and companies have developed highly specific genetic tests as LDTs to screen for and diagnose various medical conditions. According to the American Clinical Laboratory Association (ACLA), more than 11,000 labs develop and perform LDTs in the US.^9^ In 2010, the FDA announced its intent to reconsider its policy of enforcement discretion for LDTs, including most genetic tests.^7^ Predictably, the FDA’s latest action to regulate LDTs and NGS-based tests has precipitated strong reactions from industries, professional organizations, policy makers, and healthcare providers.^10^

The FDA’s final guidance offers perspective on what the agency would look for in premarket submission to determine a NGS test’s analytical validity, including “how well the tests detects presence or absence of particular genomic changes.”^11^ The guidance also outlines key considerations for designing, developing, and validating NGS-based tests used for whole exome sequencing (WES) or targeted human DNA sequencing intended to aid in the diagnosis of symptomatic individuals with suspected germline diseases. While the FDA should be applauded for their efforts to leverage NGS technology to advance precision medicine, the guidance also creates several concerns that could affect science, medicine, and patient care for years to come.

## Necessity and authority of FDA to regulate NGS-based tests

Critics question whether the FDA’s decision to regulate NGS-based tests is justified. Currently, clinical laboratories that develop and offer genetic tests are overseen by CMS through Clinical Laboratory Improvement Amendments (CLIA) regulations. Under CMS, CLIA already establishes quality and analytical validity standards for any lab conducting clinical genetic testing.^12^ Dual approval by FDA and CMS to reach the same outcome in analytical validity would be redundant and unnecessary. Rather than instituting a new FDA guidance that overlaps current CLIA regulations, a better alternative could be to expand CLIA’s current regulatory capacity to validate NGS testing, so that all labs could fulfill analytical validation requirements from one agency.

Observers of the FDA’s track record on NGS oversight have raised important concerns about the agency’s role and legitimacy in concert with existing CMS-CLIA regulations on safety, validity, and accuracy.^13,14^ Key challenges for this (and future) FDA guidance(s) on NGS-based testing will be developing fair, transparent statutes that (a) neither duplicate nor usurp existing regulations enforced by other agencies, (b) adequately validate a test’s accuracy, precision and limits of detection, and (c) foster public and private companies to continue development of NGS-based technology.

## FDA guidance threatens innovation and fair competition

Proposed requirements in the FDA’s guidance potentially create detrimental consequences to future research and development of NGS testing. The research industry comprises of small hospitals and academic research centers that are often the source of scientific innovation despite limited financial backing. The FDA’s restrictive proposals add an additional layer of regulatory requirements that small labs may not afford. FDA regulations that require costs, resources, and time are factors that could dissuade small companies from entering or continuing NGS test development, ultimately affecting the pace of scientific innovation.

Moreover, the FDA’s new guidance arguably tips the market scale in favor of large corporations with deeper financial resources. Monopolies in any industry adversely affect product development and ultimately, consumers. Research is no exception. The U.S. Supreme Court ruling *Association for Molecular Pathology v Myriad Genetics Inc*^15^ provides an interesting example. Prior to the Court’s decision, only one company had exclusive rights to offer genomic testing on the two genes linked to breast and ovarian cancer. In 2013, the Court invalidated Myriad’s patent claim to *BRCA1/2*, allowing biotechnology companies to enter the *BRCA1/2* testing market. Since the ruling, companies have markedly improved *BRCA1/2* tests with expanded capabilities, higher quality, and lower pricing.

## Expanding the scope of NGS testing

The FDA guidance article also raises questions that require further clarification and consideration for future revision. In the proposed framework, NGS-based in vitro diagnostic tests are only stipulated for symptomatic patients with suspected germline diseases, which raise several questions – Why only germline diseases? Why only just germline diseases in symptomatic patients? What about asymptomatic patients? It would be reasonable for the FDA to consider including somatic mutations in their guidance as well. At present, NGS technology is used for somatic mutation screening and diagnostic purposes in clinical settings. This has resulted in the generation of large bodies of data like National Cancer Institute’s Genomic Data Commons, which can generate somatic DNA mutation calls from DNA-Seq data of pooled tumor tissues.^16^ Somatic testing will continue to expand beyond oncology to help diagnose or treat human disease. Accordingly, the FDA should consider diseases caused by somatic mutations as another application for NGS-based testing.

In May, the FDA hosted a public forum to discuss the NGS guidance and solicit comments from attendees.^17^ When asked whether the agency would consider adding somatic mutations to their proposed framework, FDA representatives acknowledged the guidance’s narrow intended use, but did not anticipate expanding guidance to somatic mutations. Rather than developing a framework to review all mutations, the FDA sought to provide “a potential pathway, whereby NGS-based tests intended to aid in the diagnosis of suspected germline diseases, could be considered as candidates for down classification to Class II devices [and eventually 501(k) premarket exemption], since all novel tests - including those with the intended use described in the guidance - are Class III by default.”^18^ While the FDA deserves credit for their efforts to streamline the process for select LDTs (in this case, germline diseases among symptomatic patients) for premarket review, the final guidance leaves out a significant number of opportunities and clinical contexts that NGS testing could be applied.

## Future considerations

While rapid advances in genomic technology pose a challenge to fair regulatory oversight, a common goal that brings all stakeholders together is our pursuit to develop safe and accurate tests in the interest of precision medicine. At stake in this debate over the FDA guidance is patient access to useful genetic tests that can potentially guide clinical management and decision-making.

All stakeholders must continue to work together in collaboration with the FDA with a shared understanding that guidances and policies will always need modification so long as technology and medicine advance. Earlier this year, CMS took similar measures to increase patient access to NGS testing by finalizing a National Coverage Determination for Medicare beneficiaries with advanced cancer.^19^ However, neither CMS nor FDA has issued official statements concerning recent decisions made by either agency about their NGS testing policies. While the FDA’s recent public workshop and changes made from the initial draft to final guidance demonstrate the agency’s openness to continued dialogue, further research and input from other stakeholders are needed to confirm the appropriateness of the guidance *before* the FDA pushes ahead with NGS regulation according to proposed standards.

The FDA is and should remain involved in the regulation of NGS-based testing. However, too much regulation can be just as harmful as too little. Currently, NGS testing standards and guidelines are simultaneously regulated by three agencies—CMS, FDA, and the Federal Trade Commission. The roles of each agency in regulating genomic testing for precision medicine can be better organized and consolidated. Research companies across healthcare would benefit at greater extent, if a regulatory system with simpler lines of rank and duties were established. Furthermore, stringent regulations potentially harm clinical innovation and disproportionally harm small labs that must compete with big companies to submit tests for FDA approval. To maintain innovation and public trust in federal regulation, it will be necessary for the FDA to issue clear guidances and to consistently enforce regulations.

Achieving fair and transparent regulations for NGS-based testing is just a starting block for the precision medicine revolution. Even more challenging will be navigating the diverse host of economic, commercialization, insurance, and privacy concerns that follow. Other biotechnology breakthroughs, cybersecurity, and payment models are destined to become the topic of FDA guidances in the coming future - How will the FDA regulate CRISPR-Cas9 gene editing technology for clinical applications? How can the FDA incentivize private and public insurers to devise fair, adequate payment models to support NGS testing in clinical practice? What safety measures should be enforced to protect the anonymity and confidentiality of genomic data as this information is increasingly shared?

Clinical translation of genomic testing will be an ongoing, long-term process that involves balancing regulation with scientific innovation. Achieving this delicate balance will require all parties, including the government, to remain open to possibly modifying their stance and actions to collectively uphold our most important priority in precision medicine – advancing what is best for our patients.
